# Interaction network and mass spectrometry data of *Xanthomonas citri* subsp. *citri* surface proteins from differential proteomic analysis of infectious and non-infectious cells

**DOI:** 10.1016/j.dib.2016.07.054

**Published:** 2016-08-04

**Authors:** Carolina Moretto Carnielli, Juliana Artier, Julio Cezar Franco de Oliveira, Maria Teresa Marques Novo-Mansur

**Affiliations:** a Laboratório de Bioquímica e Biologia Molecular Aplicada- LBBMA, Departamento de Genética e Evolução, Universidade Federal de São Carlos, UFSCar, São Carlos, SP, Brazil; b Laboratório de Interações Microbianas, Departamento de Ciências Biológicas, Universidade Federal de São Paulo, UNIFESP, Diadema, SP, Brazil

**Keywords:** Interaction network, MS data, *Xanthomonas citri*, Surface proteome, Citrus canker

## Abstract

Here we provide the mass-spectrometry and *in silico* interaction network dataset of proteins identified on our research article on surface proteomic analysis from *Xanthomonas citri* subsp. *citri* (XAC) cells grown *in vivo* (infectious) and *in vitro* (non-infectious, control) by 2D-DIGE approach. Fluorescence labeling of proteins were performed on intact cells followed by cellular lysis and labeled spots from 2D gel differing in abundance between the two conditions (ANOVA, *p*-value<0.05) were analyzed by a nano-electrospray tandem mass spectrometry Q-Tof Ultima API mass spectrometer (MicroMass/Waters) (LC-ESI-MS/MS). This article contains raw data of proteins detected in the 79 spots analyzed by LC-ESI-MS/MS approach and also an enrichment analysis on the resulting protein–protein interaction network performed with the Integrated Interactome System (IIS) platform and Cytoscape software. The data are supplementary to our original research article, “*Xanthomonas citri* subsp. *citri* surface proteome by 2D-DIGE: ferric enterobactin receptor and other outer membrane proteins potentially involved in citric host interaction” (Carnielli et al., 2016) [1], and raw data are available via Peptide Atlas (ftp://PASS00850:ZJ7425v@ftp.peptideatlas.org/).

**Specifications Table**

**Table:** 

Subject area	*Biology*
More specific subject area	*Plant–pathogen interaction proteomics*
Type of data	*MS spectra raw files, Figure, Table*
How data was acquired	*Mass Spectrometry Liquid Chromatography: nano-electrospray tandem mass spectrometry Q-Tof Ultima API mass spectrometer system used: MicroMass/Waters*
Data format	*Raw, analyzed*
Experimental factors	*2D-DIGE proteome analysis of surface-labeled XAC cells (in vivo vs. in vitro)*
Experimental features	*XAC cells were grown in vivo (infectious) and in vitro (non-infectious) conditions and cells were fluorescently labeled previously to cell lysis. Differential spots were isolated, trypsin-digested and peptide samples were analyzed by LC-ESI-MS/MS and proteins identified by Mascot search software.*
Data source location	*Campinas and São Carlos, São Paulo State, Brazil.*
Data accessibility	*All the raw files from mass spectrometry analysis are deposited in Peptide Atlas and can be found through the PASS00850 number or by the link ftp://PASS00850:ZJ7425v@ftp.peptideatlas.org/.*

**Value of the data**

• Data were generated by a first study on surface proteome of XAC interacting with its citrus host and thus can provide additional information for XAC-host interaction studies in need of proteomic data
• *In silico* interaction analysis provides an overview of possible protein–protein interactions among XAC cells.

## 1 Data

**Fig. 1:**
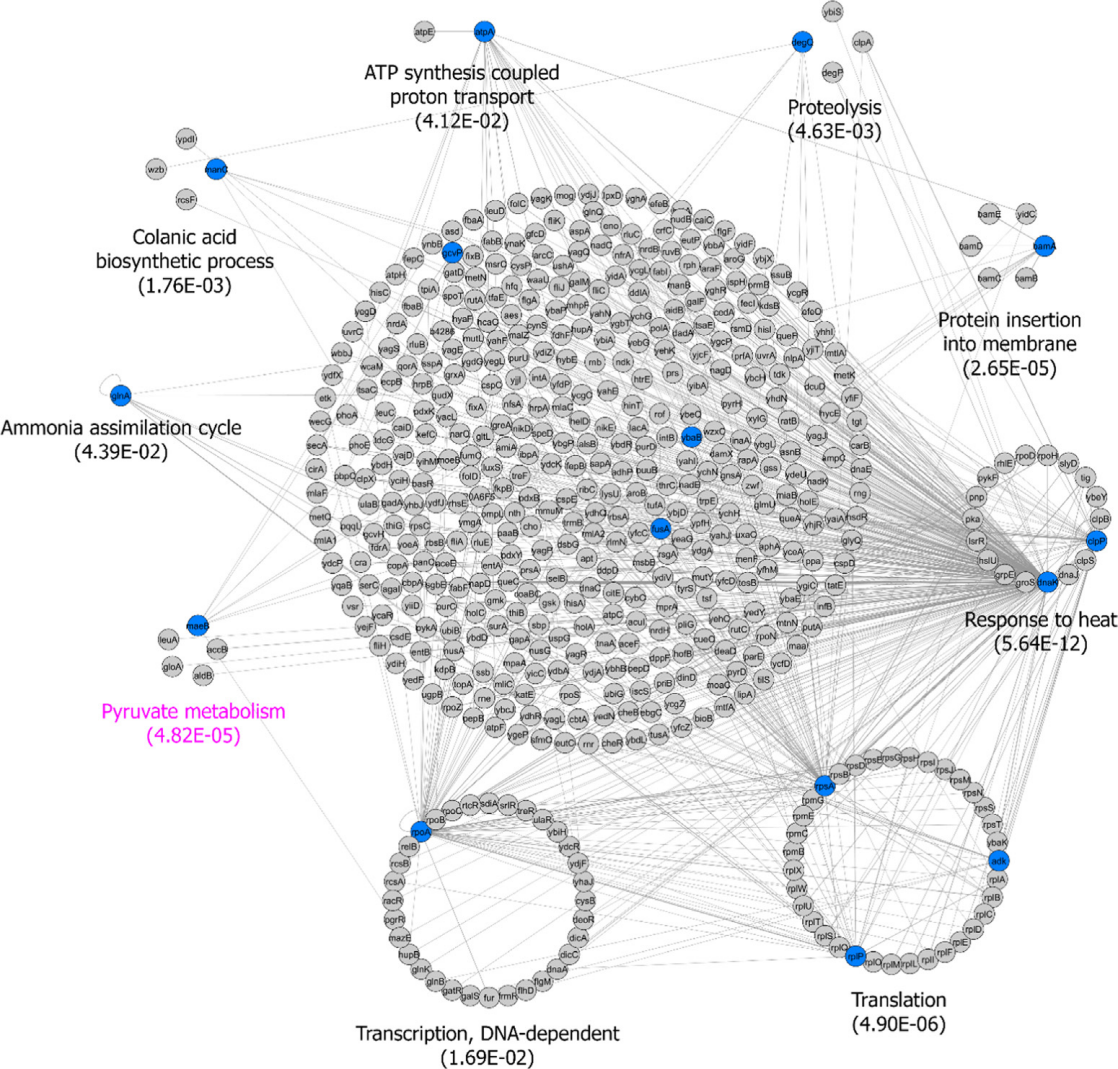
Interaction network of proteins identified in XAC spots (Table 1). The network was built using the IIS software and orthologue relationship of annotated interactions from *Escherichia coli* database. Proteins were assigned as clusters in a circle layout according to enriched biological processes (*p*-value <0.05) or enriched KEGG pathways (name written in purple color) (*p*-value <0.05). Different colors were attributed to proteins according to the input (in blue) or from the database (in gray). The resultant networks were visualized using the Cytoscape 2.8.3 software.

Data include raw files of mass spectrometry analysis of tryptic peptides of XAC surface proteins labeled with CyDyes DIGE minimal dyes. Proteins with differential abundance in cells grown *in vivo* and *in vitro* were mapped into a protein–protein interaction network (Fig. 1; Supplementary data). Information of overrepresented Gene Ontology (GO) biological processes and KEGG pathways is shown (Table 1).

## 2 Experimental design, material and methods

XAC genome strain (strain 306) was grown *in vivo* on detached *Citrus aurantifolia* leaves (infectious condition) and *in vitro* in NB medium (non-infectious condition, control), as described by Carnielli et al. [1].

### 2.1 LC-ESI-MS/MS analysis

Seventy-nine CyeDye labeled spots determined as differential by ANOVA (DeCyder software, GE Healthcare) were excised, digested with trypsin and peptide mixtures from each spot were loaded onto an analytic column C18 1.7 μm BEH 130 (100 μm×100 mm) RP-UPLC (nanoAcquity UPLC, Waters) coupled to a nano-electrospray tandem mass spectrometry Q-Tof Ultima API mass spectrometer (MicroMass/Waters). A trapping column Symmetry C18 (180 μm×20 mm) was used for desalting and sample concentration.

Data files generated by the LC-ESI-MS/MS analysis (PeptideAtlas dataset submission PASS00850) were processed using the search engine MASCOT Distiller v.2.3.2.0, 2009 (Matrix Science Ltd.) and the sequences were searched against XAC 306 genome databank (available at NCBI) using Mascot Server v.2.3.01.0 (Matrix Science Ltd.). The following parameters were used for database searches: trypsin with 1 missed cleavage allowed, mass tolerance of 0.1 Da for the precursor ions and a tolerance of 0.1 Da for the fragment ions, carbamidomethyl of cystein as fixed modification, oxidation of methionine (variable), and presence of CyDyes in lysine residues (variable).

### 2.2 Bioinformatic and network analysis

The identified proteins were submitted to an enrichment analysis by Integrated Interactome System (IIS) platform [2] using the functional annotation database of *Escherichia coli*, since *Xanthomonas* sp. does not have such database annotation. The resulting protein map was visualized on Cytoscape and nodes were assigned in clusters according to the most enriched (lowest enrichment *p*-value) biological processes or KEGG pathway (Fig. 1). Different colors were used to display proteins from the input (orthologues proteins; in blue) or from the database (in gray). Proteins without a biological process or KEGG pathway annotation are grouped in the center of the network. The annotation table for the input list is shown in Table 1.

## Figures and Tables

**Table 1:** Functional annotation analysis for the identified XAC proteins.

**ID**	**Gene symbol**	**ENSEMBL**	**SWISS-PROT**	**Protein structure (PDB)**	**Conserved domain (CDD)**	**Gene ontology (GO)**		
						**Cellular component**	**Molecular function**	**Biological process**
Q8PQW5	*glnA*	Not defined	Glutamine synthetase OS=*Methylococcus capsulatus* (strain ATCC 33,009/NCIMB 11,132/Bath) GN=glnA PE=3 SV=2	2gls GLUTAMINE SYNTHETASE	TIGR00653, GlnA, glutamine synthetase, type I	GO:0005737 cytoplasm	GO:0005524 ATP binding	GO:0006542 glutamine biosynthetic process
							GO:0004356 glutamate-ammonia ligase activity	GO:0009399 nitrogen fixation
								
Q8PQS7	*XAC0245*	Not defined	No hits found	No hits found	No hits found	Not defined	Not defined	Not defined
Q8PPZ1	*groL*	Not defined	60 kDa chaperonin OS=*Xanthomonas axonopodis* pv. *citri* (strain 306) GN=groL PE=3 SV=1	3e76 60 kDa chaperonin	PRK00013, groEL, chaperonin GroEL	GO:0005737 cytoplasm	GO:0005524 ATP binding	GO:0042026 protein refolding
Q8PNS6	*fusA*	Not defined	Elongation factor G OS=*Xanthomonas axonopodis* pv. *citri* (strain 306) GN=fusA PE=3 SV=1	4kjc elongation factor G	PRK00007, PRK00007, elongation factor G	GO:0005737 cytoplasm	GO:0005525 GTP binding	Not defined
							GO:0003924 GTPase activity	
							GO:0003746 translation elongation factor activity	
								
Q8PNR8	*rplP*	Not defined	50S ribosomal protein L16 OS=*Xanthomonas oryzae* pv. *oryzae* (strain KACC10331/KXO85) GN=rplP PE=3 SV=1	4kjb 50S ribosomal protein L16	PRK09203, rplP, 50S ribosomal protein L16	GO:0005840 ribosome	GO:0019843 rRNA binding	GO:0006412 translation
							GO:0003735 structural constituent of ribosome	
							GO:0000049 tRNA binding	
								
Q8PN59	*gcvP*	Not defined	Glycine dehydrogenase (decarboxylating) OS=*Xanthomonas axonopodis* pv. *citri* (strain 306) GN=gcvP PE=3 SV=1	4lhd Glycine dehydrogenase [decarboxylating]	PRK05367, PRK05367, glycine dehydrogenase	Not defined	GO:0004375 glycine dehydrogenase (decarboxylating) activity	GO:0019464 glycine decarboxylation via glycine cleavage system
							GO:0030170 pyridoxal phosphate binding	
								
Q8PNG1	*XAC1110*	Not defined	Nucleoid-associated protein XAC1110 OS=*Xanthomonas axonopodis* pv. *citri* (strain 306) GN=XAC1110 PE=3 SV=1	1pug Hypothetical UPF0133 protein ybaB	PRK00153, PRK00153, hypothetical protein	GO:0043590 bacterial nucleoid	GO:0003677 DNA binding	Not defined
						GO:0005737 cytoplasm		
								
Q8PNI5	*clpP*	Not defined	ATP-dependent Clp protease proteolytic subunit OS=*Xanthomonas oryzae* pv. *oryzae* (strain KACC10331/KXO85) GN=clpP PE=3 SV=1	2fzs ATP-dependent Clp protease proteolytic subuni	PRK00277, clpP, ATP-dependent Clp protease proteolytic subunit	GO:0005737 cytoplasm	GO:0004252 serine-type endopeptidase activity	Not defined
Q8PNP2	*mopB*	Not defined	Outer membrane porin F OS=*Pseudomonas aeruginosa* (strain ATCC 15,692/PAO1/1C/PRS 101/LMG 12,228) GN=oprF PE=1 SV=1	No hits found	cd07185, OmpA_C-like, Peptidoglycan binding domains similar to the C-terminal domain of outer-membrane protein OmpA	GO:0009279 cell outer membrane	GO:0005509 calcium ion binding	Not defined
						GO:0016021 integral to membrane		
						GO:0005886 plasma membrane		
								
Q8PNP8	*mdh*	Not defined	Malate dehydrogenase OS=*Xanthomonas axonopodis* pv. *citri* (strain 306) GN=mdh PE=3 SV=1	1b8v PROTEIN (MALATE DEHYDROGENASE)	PRK05442, PRK05442, malate dehydrogenase	Not defined	GO:0030060 L-malate dehydrogenase activity	GO:0044262 cellular carbohydrate metabolic process
								GO:0006108 malate metabolic process
								GO:0006099 tricarboxylic acid cycle
								
Q8PK77	*rpsA*	Not defined	30S ribosomal protein S1 OS=*Pseudomonas aeruginosa* (strain ATCC 15,692/PAO1/1C/PRS 101/LMG 12,228) GN=rpsA PE=3 SV=1	2khi 30S ribosomal protein S1	PRK06299, rpsA, 30S ribosomal protein S1	GO:0005840 ribosome	GO:0003723 RNA binding	GO:0006412 translation
							GO:0003735 structural constituent of ribosome	
								
Q8PMB0	*dnaK*	Not defined	Chaperone protein DnaK OS=*Xanthomonas axonopodis* pv. *citri* (strain 306) GN=dnaK PE=3 SV=1	2kho Heat shock protein 70	PRK00290, dnaK, molecular chaperone DnaK	Not defined	GO:0005524 ATP binding	GO:0006457 protein folding
								GO:0006950 response to stress
								
Q8PMC2	*XAC1509*	Not defined	No hits found	No hits found	No hits found	Not defined	Not defined	Not defined
Q8PML3	*oma*	Not defined	Outer membrane protein assembly factor BamA OS=*Edwardsiella ictaluri* (strain 93–146) GN=bamA PE=3 SV=1	4k3b Outer membrane protein assembly factor BamA	TIGR03303, OM_YaeT, outer membrane protein assembly complex, YaeT protein	GO:0009279 cell outer membrane	Not defined	GO:0043165 Gram-negative-bacterium-type cell outer membrane assembly
						GO:0016021 integral to membrane		GO:0051205 protein insertion into membrane
						GO:0005886 plasma membrane		
								
Q8PMV4	*mucD*	Not defined	Probable periplasmic serine endoprotease DegP-like OS=*Pseudomonas savastanoi* pv. *phaseolicola* (strain 1448A/Race 6) GN=mucD PE=3 SV=1	3otp Protease do	TIGR02037, degP_htrA_DO, periplasmic serine protease, Do/DeqQ family	Not defined	GO:0004252 serine-type endopeptidase activity	Not defined
Q8PJ70	*oar*	Not defined	No hits found	No hits found	pfam13620, CarboxypepD_reg, Carboxypeptidase regulatory-like domain	GO:0016020 membrane	GO:0004872 receptor activity	Not defined
							GO:0005215 transporter activity	
								
Q8PJ69	*XAC2673*	Not defined	No hits found	No hits found	No hits found	Not defined	Not defined	Not defined
Q8PJ68	*XAC2674*	Not defined	UPF0056 inner membrane protein YhgN OS=*Shigella flexneri* GN=yhgN PE=3 SV=1	No hits found	COG2095, MarC, Multiple antibiotic transporter [Intracellular trafficking and secretion]	GO:0016021 integral to membrane	Not defined	Not defined
Q8PI27	*iroN*	Not defined	No hits found	No hits found	TIGR01782, TonB-Xanth-Caul, TonB-dependent receptor	GO:0009279 cell outer membrane	GO:0004872 receptor activity	Not defined
							GO:0005215 transporter activity	
								
Q8PHT1	*bfeA*	Not defined	No hits found	No hits found	cd01347, ligand_gated_channel, TonB dependent/Ligand-Gated channels are created by a monomeric 22 strand (22,24) anti-parallel beta-barrel	GO:0009279 cell outer membrane	GO:0004872 receptor activity	Not defined
							GO:0005215 transporter activity	
								
Q8PHT0	*bfeA*	Not defined	No hits found	No hits found	cd01347, ligand_gated_channel, TonB dependent/Ligand-Gated channels are created by a monomeric 22 strand (22,24) anti-parallel beta-barrel	GO:0009279 cell outer membrane	GO:0004872 receptor activity	Not defined
							GO:0005215 transporter activity	
								
Q8PGZ2	*maeB*	Not defined	NADP-dependent malic enzyme OS=*Escherichia coli* (strain K12) GN=maeB PE=1 SV=1	2dvm 439aa long hypothetical malate oxidoreductase	PRK07232, PRK07232, bifunctional malic enzyme oxidoreductase/phosphotransacetylase	GO:0005829 cytosol	GO:0004471 malate dehydrogenase (decarboxylating) activity	GO:0006108 malate metabolic process
							GO:0004473 malate dehydrogenase (oxaloacetate-decarboxylating) (NADP+) activity	
							GO:0030145 manganese ion binding	
							GO:0051287 NAD binding	
							GO:0016746 transferase activity, transferring acyl groups	
								
Q8PH16	*btuB*	Not defined	No hits found	No hits found	cd01347, ligand_gated_channel, TonB dependent/Ligand-Gated channels are created by a monomeric 22 strand (22,24) anti-parallel beta-barrel	GO:0009279 cell outer membrane	GO:0004872 receptor activity	Not defined
							GO:0005215 transporter activity	
								
Q8PH23	*adk*	Not defined	Adenylate kinase OS=*Xanthomonas campestris* pv. *vesicatoria* (strain 85-10) GN=adk PE=3 SV=1	1p4s Adenylate kinase	PRK00279, adk, adenylate kinase	GO:0005737 cytoplasm	GO:0004017 adenylate kinase activity	GO:0044209 AMP salvage
							GO:0005524 ATP binding	
								
Q8PH89	*fhuE*	Not defined	FhuE receptor OS=*Escherichia coli* (strain K12) GN=fhuE PE=1 SV=2	3efm Ferric alcaligin siderophore receptor	COG4773, FhuE, Outer membrane receptor for ferric coprogen and ferric-rhodotorulic acid [Inorganic ion transport and metabolism]	GO:0009279 cell outer membrane	GO:0005506 iron ion binding	Not defined
							GO:0004872 receptor activity	
							GO:0015343 siderophore transmembrane transporter activity	
								
Q8PHB5	*XAC3344*	Not defined	Probable fructose-bisphosphate aldolase class 1 OS=*Xanthomonas axonopodis* pv. *citri* (strain 306) GN=XAC3344 PE=3 SV=1	3mmt Fructose-bisphosphate aldolase	cd00948, FBP_aldolase_I_a, Fructose-1,6-bisphosphate aldolase	Not defined	GO:0004332 fructose-bisphosphate aldolase activity	GO:0006096 glycolysis
Q8PFD5	*iroN*	Not defined	No hits found	No hits found	TIGR01782, TonB-Xanth-Caul, TonB-dependent receptor	GO:0009279 cell outer membrane	GO:0004872 receptor activity	Not defined
							GO:0005215 transporter activity	
								
Q8PFH2	*argI*	Not defined	Arginase OS=*Brucella suis* biovar 1 (strain 1330) GN=arcB PE=3 SV=1	5cev PROTEIN (ARGINASE)	cd09989, Arginase, Arginase family	Not defined	GO:0004053 arginase activity	GO:0006525 arginine metabolic process
							GO:0046872 metal ion binding	
								
Q8PG19	*XAC3802*	Not defined	No hits found	No hits found	No hits found	Not defined	Not defined	Not defined
Q8PGG5	*atpA*	Not defined	ATP synthase subunit alpha OS=*Xanthomonas campestris* pv. *vesicatoria* (strain 85-10) GN=atpA PE=3 SV=1	3oaa ATP synthase subunit alpha	PRK09281, PRK09281, F0F1 ATP synthase subunit alpha	GO:0005886 plasma membrane	GO:0005524 ATP binding	GO:0015991 ATP hydrolysis coupled proton transport
						GO:0045261 proton-transporting ATP synthase complex, catalytic core F(1)	GO:0046933 proton-transporting ATP synthase activity, rotational mechanism	GO:0042777 plasma membrane ATP synthesis coupled proton transport
							GO:0046961 proton-transporting ATPase activity, rotational mechanism	
								
Q8PGN6	*xanB*	Not defined	Xanthan biosynthesis protein XanB OS=*Xanthomonas campestris* pv. *campestris* (strain ATCC 33,913/DSM 3586/NCPPB 528/LMG 568/P 25) GN=xanB PE=3 SV=1	2x65 MANNOSE-1-PHOSPHATE GUANYLYLTRANSFERASE	TIGR01479, GMP_PMI, mannose-1-phosphate guanylyltransferase/mannose-6-phosphate isomerase	Not defined	GO:0016853 isomerase activity	GO:0000271 polysaccharide biosynthetic process
							GO:0016779 nucleotidyltransferase activity	
								
Q8PGY7	*XAC3475*	Not defined	No hits found	No hits found	pfam13531, SBP_bac_11, Bacterial extracellular solute-binding protein	Not defined	Not defined	Not defined
Q8PER7	*XAC4273*	Not defined	No hits found	No hits found	No hits found	GO:0016020 membrane	GO:0030246 carbohydrate binding	Not defined
							GO:0004872 receptor activity	
							GO:0005215 transporter activity	
								
Q8PER6	*XAC4274*	Not defined	No hits found	No hits found	No hits found	GO:0016020 membrane	GO:0030246 carbohydrate binding	Not defined
							GO:0004872 receptor activity	
							GO:0005215 transporter activity	
								
Q8NL22	*tufA*	Not defined	Elongation factor Tu OS=*Xanthomonas campestris* pv. *vesicatoria* (strain 85-10) GN=tuf1 PE=3 SV=1	4g5g Elongation factor Tu 1	PRK00049, PRK00049, elongation factor Tu	GO:0005737 cytoplasm	GO:0005525 GTP binding	Not defined
							GO:0003924 GTPase activity	
							GO:0003746 translation elongation factor activity	
								
P0A0Y2	*rpoA*	Not defined	DNA-directed RNA polymerase subunit alpha OS=*Xanthomonas oryzae* pv. *oryzae* (strain KACC10331/KXO85) GN=rpoA PE=3 SV=1	4kn7 DNA-directed RNA polymerase subunit alpha	PRK05182, PRK05182, DNA-directed RNA polymerase subunit alpha	Not defined	GO:0003677 DNA binding	GO:0006351 transcription, DNA-dependent
							GO:0003899 DNA-directed RNA polymerase activity	

## References

[1] Carnielli C.M., Artier J., de Oliveira J.C.F., Novo-Mansur M.T.M. (2016). *Xanthomonas citri* subsp. *citri* surface proteome by 2D-DIGE: ferric enterobactin receptor and other outer membrane proteins potentially involved in citric host interaction. J. Proteom..

[2] Carazzolle M.F., de Carvalho L.M., Slepicka H.H., Vidal R.O., Pereira G.A., Kobarg J. (2014). IIS--Integrated Interactome System: a web-based platform for the annotation, analysis and visualization of protein-metabolite-gene-drug interactions by integrating a variety of data sources and tools. PLoS One.

